# Mammalian cell expression, purification, crystallization and microcrystal data collection of autotaxin/ENPP2, a secreted mammalian glycoprotein

**DOI:** 10.1107/S1744309110032938

**Published:** 2010-08-31

**Authors:** Jens Hausmann, Evangelos Christodoulou, Mobien Kasiem, Valeria De Marco, Laurens A. van Meeteren, Wouter H. Moolenaar, Danny Axford, Robin L. Owen, Gwyndaf Evans, Anastassis Perrakis

**Affiliations:** a Division of Biochemistry B8, The Netherlands Cancer Institute, Plesmanlaan 121, 1066 CX Amsterdam, The Netherlands; b Division of Cell Biology B6, The Netherlands Cancer Institute, Plesmanlaan 121, 1066 CX Amsterdam, The Netherlands; c Diamond Light Source, Harwell Science and Innovation Campus, Didcot OX11 0DE, England

**Keywords:** microcrystals, mammalian cell expression, autotaxin, ENPP2

## Abstract

Autotaxin (ATX or ENPP2) is a secreted glycosylated mammalian enzyme that exhibits lysophospholipase D activity, hydrolyzing lysophosphatidylcholine to the signalling lipid lysophosphatidic acid. ATX is an ∼100 kDa multi-domain protein encompassing two N-terminal somatomedin B-like domains, a central catalytic phosphodiesterase domain and a C-terminal nuclease-like domain. Protocols for the efficient expression of ATX from stably transfected mammalian HEK293 cells in amounts sufficient for crystallographic studies are reported. Purification resulted in protein that crystallized readily, but various attempts to grow crystals suitable in size for routine crystallographic structure determination were not successful. However, the available micrometre-thick plates diffracted X-rays beyond 2.0 Å resolution and allowed the collection of complete diffraction data to about 2.6 Å resolution. The problems encountered and the current advantages and limitations of diffraction data collection from thin crystal plates are discussed.

## Abstract 2

Autotaxin, a four-domain ∼100 kDa mammalian glycoprotein, was expressed in stably transfected mammalian cells, purified from the medium and crystallized. Diffraction data from micrometre-thick crystal plates were collected on various European synchrotron beamlines and are presented and analysed.

## 1. Introduction

Autotaxin (ATX), the second member of the ectonucleotide phosphodiesterase/pyrophosphatase (ENPP) family, is a secreted ∼100 kDa glycoprotein (Stracke *et al.*, 1992 ▶). ATX/ENPP2 is classified as a phosphoric diester hydrolase (EC 3.1.4.–) that displays alkylglycero­phosphoethanolamine phosphodiesterase (lysophospho­lipase D) activity (Umezu-Goto *et al.*, 2002 ▶; EC 3.1.4.39). Autotaxin converts extracellular lysophosphatidylcholine (LPC) to lysophos­phatidic acid (LPA), a signalling phospholipid that acts on at least six distinct G-­protein-coupled receptors (LPA1–6) and elicits a great variety of both short-term and long-term cellular responses (van Meeteren & Moolenaar, 2007 ▶). ATX and LPA signalling have been strongly implicated in tumour progression and metastasis (Mills & Moolenaar, 2003 ▶), as well as in angiogenesis (Fotopoulou *et al.*, 2010 ▶; Tanaka *et al.*, 2006 ▶; van Meeteren *et al.*, 2006 ▶). As such, ATX holds great promise as a therapeutic target (Albers *et al.*, 2010 ▶).

ATX harbours a central catalytic domain that is similar to bacterial phosphodiesterases and alkaline phosphatases. This domain is flanked N-terminally by two Cys-rich somatomedin B-like (SMB) domains and C-terminally by a nuclease-like domain. The intact secreted protein is required for activity and glycosylation has been shown to be crucial for function (Jansen *et al.*, 2007 ▶). We therefore set out to produce ATX in sufficient amounts for crystallo­graphic studies.

Protocols for the overexpression of secreted proteins in mammalian cell lines and amounts suitable for crystallographic studies have been established as part of the SPINE consortium (Aricescu *et al.*, 2006 ▶). We adapted these protocols and extended them to the use of stably transfected cell lines.

Although ATX was easy to crystallize once produced in homogenous form and good quantity, we could only obtain plate-like microcrystals. Despite older (Cusack *et al.*, 1998 ▶; Perrakis *et al.*, 1999 ▶) and more recent advances (Cherezov *et al.*, 2009 ▶; Moukhametzianov *et al.*, 2008 ▶; Sanishvili *et al.*, 2008 ▶; Schneider, 2008 ▶) in microcrystallography, data collection from plate-like microcrystals presented a challenge, which we discuss here.

## 2. Experimental procedures and results

### 2.1. Construction of a stable cell line expressing ATX

An rATX construct retaining only one glycosylation site essential for activity (Jansen *et al.*, 2007 ▶) was amplified by PCR from plasmid DNA (using 5′-TGGTACCGCCACCATGGCAAGACAAGGCTG­TCTC-3′ and 5′-ACCGGTAATCTCGCTCTCATATGT-3′ primers) and the reaction product was cut with *Asp*718I and *Age*I restriction enzymes and cloned into the plasmid pcDNA3.1/V5-His (Invitrogen) in order to introduce a C-terminal His_6_ tag. The rATX-His gene was amplified by PCR (using 5′-TGGTACCGCCACCATGGCAAGAC­AAGGCTGTCTC-3′ and 5′-GGATCCTCAATGGTGATGGTGA­TG-3′ primers), cut with *Asp*718I and *Pin*AI restriction enzymes and cloned into the plasmid pcDNA5/FRT. Restriction digests were performed for 1 h at 310 K. PCR reactions were performed using *Pfu* polymerase (Stratagene; catalogue No. 600353-51) in 50 µl reaction volumes using 30 PCR cycles (denaturation at 369 K for 30 s, annealing at 328 K for 30 s and 345 K for 120 s). All other conditions used were according to the manufacturer’s instructions and used the reaction buffers supplied by the manufacturer. The final clone was verified by sequencing.

HEK 293 Flp-In cells (Invitrogen) were grown in 10 cm tissue-culture dishes in DMEM medium (Invitrogen; catalogue No. 41966-052) containing 10% FCS (Sigma; catalogue No. 8200496835) and 100 µg ml^−1^ Zeocin (Invitrogen; catalogue No. 45-0430). On the day of transfection, the cells (80–90% confluent) were washed with medium without Zeocin and co-transfected with 2 µg pcDNA5/FRT-rATX-His and 18 µg pOG44 (Invitrogen; catalogue No. K6010-01) using lipofectamine (Invitrogen; catalogue No. 18324012). Next day, the cells were washed using fresh medium without antibiotics. The following day, the cells were split to ∼20% confluence in fresh medium containing 100 µg ml^−1^ hygromycin B (Invitrogen; catalogue No. 10687-010) and allowed to grow for approximately 10 d until foci could be identified. Six foci were picked and expanded in T175 tissue-culture flasks in DMEM medium containing 10% FCS and 100 µg ml^−1^ hygromycin B to verify resistance in hygromycin B. The cells were flash-frozen in liquid nitrogen for future use.

### 2.2. Large-scale expression

For large-scale expression, cells were first grown in T175 tissue-culture flasks in DMEM medium containing 10% FCS and 100 µg ml^−1^ hygromycin B. The cells were grown to 80–90% confluence, washed twice with fresh DMEM medium and transferred to roller bottles (Greiner Bio-One; catalogue No. 681070). Typically, a single T175 flask was used to inoculate one roller bottle and the cells were cultured for 4 d after transfer into 125 ml DMEM containing 10% FCS. The medium was changed to 125 ml DMEM supplemented with 2 m*M* glutamate (GIBCO; catalogue No. 25030-123) and the cells were left to express protein for 4 d. The medium was collected and the cells were supplemented with fresh medium and left to express protein for a further 4 d.

### 2.3. Purification of secreted ATX

The medium was collected and filtered through a 0.45 µm bottle-top filter. The filtered medium was subsequently applied at a flow rate of 10–15 ml min^−1^ onto an ∼10 ml POROS-20 MC column that had been pre-loaded with Ni^2+^. The column was washed with eight to ten column volumes of buffer *A* (20 m*M* Tris–HCl pH 8.0, 150 m*M* NaCl and 10–20 m*M* imidazole). The protein was eluted with a short (2–3 column volumes) linear gradient to buffer *A* containing 750 m*M* imidazole. The rATX fractions were mostly pure, with the exception of an ∼200 kDa band. To remove this band, the pooled fractions were concentrated and applied onto a Superose 6 10/30 size-exclusion column in buffer *A*. rATX eluted as a single symmetric peak at a column volume corresponding to a monomer of ∼100 kDa. The peak fractions were pooled and concentrated to ∼3–4 mg ml^−1^ for crystallization.

Concentration posed a serious practical problem, as rATX would ‘stick’ in most commonly used centrifugal filters, preventing con­centration. We initially resorted to dialysis against 30% PEG 10 000 to concentrate the protein. However, the use of Ultra-15 (Amicon; 10 kDa molecular-weight cutoff) centrifugal filters allowed more efficient concentration later in the course of the project. Preparation of eight roller bottles typically allowed 2–3 mg of protein to be produced.

### 2.4. Crystallization

rATX was initially crystallized by vapour diffusion in nanodrops using the protocols summarized in Newman *et al.* (2005 ▶). Crystals in the shape of microscopic plates appeared in many conditions from the PACT screen (manufactured by Qiagen and described by Newman *et al.*, 2005 ▶). All conditions that produced initial hits contained PEG by definition and, given the highly redundant nature of the PACT screen, hits appeared in about ten drops; the conditions giving the best crystals were B5 [0.1 *M* MIB buffer pH 8, 25%(*w*/*v*) PEG 1500], B6 [0.1 *M* MIB buffer pH 9, 25%(*w*/*v*) PEG 1500] and E11 [0.2 *M* sodium citrate, 20%(*w*/*v*) PEG 3350]. Various attempts to improve the size of these crystals failed; the largest crystals that we were able to grow using these conditions were plates of several tens of a micrometre across but of sub-micrometre thickness at best.

After additive screening, octanoyl-*N*-methylglucamide (MEGA-8) appeared to have a positive effect on crystal size. Crystallization in very large drops with a total volume of 10 µl produced plate-like crystals of excellent morphology with a smallest dimension estimated to be just above 1 µm (Table 1 ▶).

Following optimization of the rATX purification protocol, additional optimization showed that micrometre-thick crystals could be grown, without the need for detergent or large drops, in standard SBS-format crystallization plates with drops of 300 nl total volume using a Mosquito liquid-handling robot. The presence of phosphate ions at a concentration of 20–200 m*M* was crucial for the formation of these crystals, which appeared over a wide pH range and had a morphology that was identical to those produced in the presence of detergent (Table 1 ▶). These crystals formed with or without seeding, although seeding accelerated crystal formation.

In both crystallization protocols a higher concentration of protein correlated well with increased crystal size. However, the practical yields of protein did not allow concentration above a certain limit, which was maximally ∼5 mg ml^−1^.

Although we successfully produced SeMet-substituted protein in the stable HEK 293 cell line, yields were significantly lower owing to the toxicity of selenomethionine, which precluded sufficient con­centration of the protein for the growth of crystals of size similar to those of the native protein.

**Figure 1:**
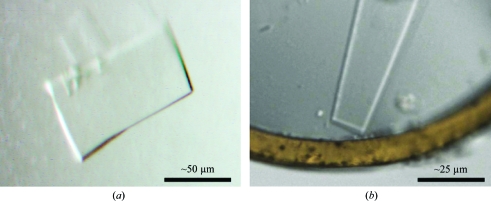
The crystals used for data collection. (*a*) ‘Crystal 2’ exposed at the SLS in the crystallization drop. (*b*) ‘Crystal 3’ exposed at Diamond as seen at the beamline mounted in a loop and flash-frozen.

Crystals were transferred to a solution typically containing 20–25% glycerol in addition to the mother liquor and mounted in loops. All crystals were vitrified by plunging them in liquid nitrogen, mounted in SPINE pins and transferred into SPINE/ESRF pucks for shipment and data collection at the synchrotron (Beteva *et al.*, 2006 ▶). During the course of about two years, a couple of thousand crystals were mounted and about a third of these attempts gave single crystals in the loop. Roughly 500 crystals were tested for diffraction. About 30 data sets were collected in total and the best data are presented in this paper. The crystals used to collect these data are shown in Fig. 1 ▶.

### 2.5. Collection of diffraction data and analysis

We discuss the measurement of three data sets: two were collected from crystals grown from very large crystallization drops (10 µl), one using a microbeam (10 × 10 µm) and one using a typical sized beam (40 × 100 µm); the third data set was collected from crystals grown in small crystallization drops (0.3 µl).

#### 2.5.1. SLS data collection with a microdiffractometer

**Figure 2:**
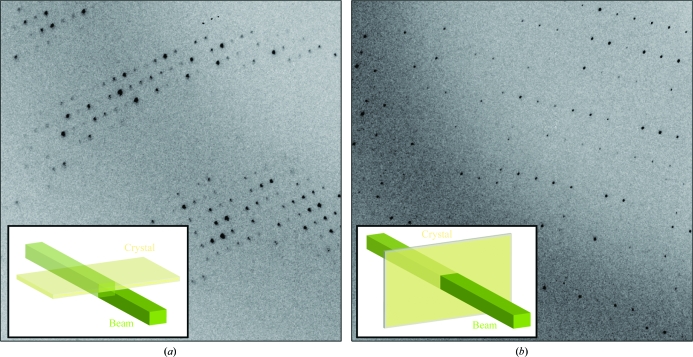
Setups for diffraction data collection (with the beam shown in green and the crystal in yellow) and the corresponding sample diffraction images, illustrating the differences bwtween the (*a*) ‘edge-on’and (*b*) ‘face-on’ orientations. The sizes of the beam and the crystal in the diagram are approximate.

A good-quality crystal was obtained after extensive screening on the SLS X06SA beamline (PX1) in a setup equipped with the EMBL/ESRF microdiffractometer (Perrakis *et al.*, 1999 ▶) and a 10 µm beam-defining aperture. Based on our experience with previous crystals, we knew that they had a limited lifetime. The longer dimension of the crystal was oriented along the rotation axis to allow translation between successive wedges and to minimize radiation damage. The first wedge of 30° was collected ‘edge-on’, while the last 20° were collected ‘face-on’. It can be seen from Figs. 2 ▶(*a*) and 2 ▶(*b*) that in the ‘edge-on’ orientation diffraction is strong but the spots are imperfect owing to crystal imperfections along the long plate axis fully bathed in the beam, while in the ‘face-on’ orientation diffraction is significantly weaker but the spots are very small and round-shaped with clear edges since only a very limited volume of the crystal is exposed. This strategy resulted in a data set of good quality; the spot intensities were integrated in *MOSFLM* using the ‘RESOLUTION CUTOFF 1.0’ keyword that limits the radial integration area in each image to the resolution bin in which the average *I*/σ(*I*) falls below 1.0. The resulting data set was complete to about 3.2 Å resolution and contained useful data to 2.6 Å resolution (Table 2 ▶).

#### 2.5.2. SLS data collection with a ‘typical’ beam and a Pilatus detector

At a later data-collection trip, a crystal of similar quality but possibly exceeding 1 µm in thickness was identified. At this time we were using the SLS ‘high-resolution diffractometer’ with a beam of approximately 40 × 100 µm and we were aware that we were lacking ‘face-on’ data from the previous data set. We were only able to collect 25° of data before the crystal suffered from radiation damage. The data-quality statistics for this ∼50% complete data set (Table 2 ▶) demonstrated that the crystal plates were of excellent quality, with minimal distortion around their surface. We observed that our crystal could withstand a higher X-ray dose in the shutter-less data-collection mode enabled by the Pilatus 6M detector (Broennimann *et al.*, 2006 ▶), than in the same beam with the same exposure and a normal CCD detector.

#### 2.5.3. A combined native data set and obstacles in collecting an SeMet data set

Combining the above data, we obtained a data set to 2.6 Å resolution (Table 3 ▶). This data set had improved completeness, specifically in the higher resolution shell (69.7% compared with 40.2%), owing to the contribution of the 25° wedge contributed by the second crystal in the ‘face-on’ orientation. Although this region was collected with the microbeam for the first crystal, these ‘face-on’ images contained diffraction to much lower resolution.

This data set was used for molecular replacement using a bacterial phosphodiesterase domain (Zalatan *et al.*, 2006 ▶) homologous to one of the four domains of ATX (with 28% identity) as a search model. Although a clear solution was easily obtained in *Phaser* (McCoy *et al.*, 2007 ▶), efforts to refine this model and complete the structure were unsuccessful. The free *R* factor did not decrease and the electron-density maps did not show any features for either the differences between the bacterial and mammalian sequences or the three domains that were not present in the search model (∼60% of the molecule).

Therefore, we focused our attention on obtaining an SeMet data set, a quest that failed. This effort was hampered by a lack of the SeMet protein required to obtain sufficiently large crystals. The crystals did not exceed 1 µm in thickness and the other two plate dimensions were typically less than 40 µm. We were thus unable to collect a redundant and high-quality data set even to a modest resolution of about ∼3.5 Å. Minimizing the exposure in order to increase the lifetime led to data that were too weak. While the SAD data that we were able to collect from a few small crystals were of reasonable quality, the redundancy was too low (anomalous redundancy of 3.7 for our best data set) to generate a sufficient anomalous signal-to-noise ratio (the anomalous correlation dropped below 30% at ∼7 Å resolution for the best data set). Attempts to combine data sets from different crystals did not produce an improved anomalous signal-to-­noise ratio. Soaking experiments proved physically difficult to perform owing to the small crystal size and did not result in usable data.

#### 2.5.4. Data collection on the Diamond I24 microfocus beamline using variable beam size

During our attempts to obtain an SeMet crystal from nanodrops for diffraction studies, we produced native crystals with some alterations in the crystallization protocol (Table 1 ▶). The size of these crystals was similar to that of the previous crystals, despite being grown from nanodrops, and they were used to test data-collection protocols for subsequent SeMet data collection at I24, a tuneable microfocus beamline that at the time was in the late stages of commissioning at Diamond Light Source. The versatile optical design of I24 (Evans *et al.*, 2006 ▶) allows the use of three beam sizes, a microbeam of 8 × 8 µm, a medium-sized beam of 15 × 20 µm and a larger 30 × 50 µm beam (v × h). As changes in beam size are achieved *via* the refocusing of mirrors rather than through the use of apertures or defining slits, the total flux at the sample position of ∼10^12^ photons s^−1^ is preserved for each of these different-sized beams. I24 was designed specifically to allow the matching of X-ray beam properties to sample properties and in the simplest case this means that where possible the beam size is matched to the projected dimensions of the crystal along the beam axis.

For these crystals the beam size was adjusted accordingly for different crystal orientations and this allowed a data set of good quality and completeness to be collected from a single microcrystal.

## 3. Discussion

### 3.1. Protein production in stably transfected mammalian cells for crystallographic studies

We have expressed a mammalian glycoprotein in stably transfected HEK 293 cells. Producing a stable cell line with the Flp-In system is straightforward and allows the routine production of protein without the need for transfection, minimizing the routine costs (the cost of large DNA preparations for transfection of large volumes of cell culture as well as the cost of transfection media are eliminated) as well as the uncertainty and heterogeneity of transfection success. Production in roller bottles allows a straightforward scale-up of the procedure at a low cost since the use of FCS is eliminated in the final stages. Loading of the medium containing the secreted protein onto a fast flow resin allowed much faster protein purification without prior concentration. Additionally, screening commercial products to find a device that allowed centrifugal protein concentration was important in order to be able to concentrate the protein further, faster and in smaller volumes and enabled more efficient crystallization screening. Finally, we were able to use nanocrystallization vapour-diffusion experiments in 0.3 µl drops, yielding excellent quality small crystals similar to these obtained in 10 µl drops. This is another case in a largely anecdotal line of evidence that argues that drop size does not correlate well with crystal size and quality.

### 3.2. Limitations of using micrometre-thick plate-like crystals

Crystallization screening using the vapour-diffusion method in nanodrops allowed the production of very small plate-like crystals. Subsequent optimization in very large drops (10 µl total) in the presence of detergent or subsequently also in nanodrops (0.3 µl) allowed the formation of micrometre-thick crystals that were suitable for collecting diffraction data to 2.6 Å resolution.

Despite recent excellent advances in instrumentation, the manipulation of very thin plate-shaped crystals presents clear challenges that need to be resolved. Since plates have a preferred orientation in mounting loops, they are invisible in ‘edge-on’ orientation, calling for grid-scan procedures (Aishima *et al.*, 2010 ▶; Bowler *et al.*, 2010 ▶; Song *et al.*, 2007 ▶), such as that available at I24, to determine the ideal centring by diffraction in that orientation.

**Figure 3:**
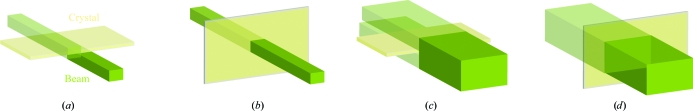
A schematic diagram illustrating the benefits and problems of small and large beams (shown in green) for plate-like crystals (shown in yellow): (*a*) thin plate ‘edge-on’ with microbeam (good), (*b*) thin plate ‘face-on’ with microbeam (bad), (*c*) thin plate ‘edge-on’ with large beam (bad) and (*d*) thin plate ‘face-on’ with large beam (good).

Furthermore, while a microbeam is ideal for collecting data in the ‘edge-on’ orientation, a larger size beam is preferable ‘face-on’, as illustrated in Fig. 3 ▶. As an example, consider two possible beam sizes, a microbeam of 10 × 10 µm and a more common beam size of 100 × 50 µm, being used to measure data from a typical rATX crystal of dimensions 100 × 50 × 1 µm. Clearly, neither situation is optimal. In the ‘edge-on’ orientation a microbeam is best (Fig. 3 ▶ *a*). With such a thin crystal, the maximal volume is exposed in the ‘edge-on’ orientation: for the dimensions given above, in this orientation a volume of ∼500 µm^3^ is exposed (1 µm crystal thickness × 10 µm of crystal along the long axis across the beam, which is limited by the beam size, × 50 µm of the crystal’s other long axis along the beam). In the ‘face-on’ orientation only ∼100 µm^3^ of crystal volume would be exposed to the micro-beam (Fig. 3 ▶ *b*; 1 µm crystal thickness × 10 µm × 10 µm of crystal limited by the beam size in both long crystal dimensions). However, after a few degrees of rotation one can use a larger beam and finally in the ‘face-on’ orientation a large beam matching the plate size is best suited (Fig. 3 ▶ *d*; 1 µm crystal thickness × 100 µm × 50 µm of crystal along the long axes across the beam) and would result in ∼5000 µm^3^ of crystal being exposed. However, the large beam would result in a too high a background in the ‘edge-on’ orientation, as effectively only 2% of the beam would hit the crystal (Fig. 3 ▶ *c*). In our opinion, variable beam sizes in which the beam size adapts dynamically during data collection could have possibly improved data collection from these crystals.

The availability of a variable beam size at I24 allowed data collection from a single plate-shaped crystal even though only three discrete sizes were offered at the time. Use of the grid-scan technique to centre the ‘edge-on’ orientation and the sub­sequent use of variable beam sizes resulted in data being measured from a single crystal that were of a quality comparable to those obtained from two crystals on the SLS X06SA beamline using two different microdiffractometers. At the time of these experiments only three discrete beam sizes were available at I24, but the design goal for this beamline is continuous and independent variability of beam size in both directions so as to permit optimal adaptation of the beam characteristics to the size and orientation of the sample.

We argue here that an X-ray beam that could be changed in size every few degrees throughout data collection or software that would allow three-dimensional data-collection strategies utilizing all of the volume of thin plate-shaped crystals could allow more efficient data collection. If the available beam size matches the smallest crystal dimension, microcrystals, in a way, present the lesser challenge for data collection: the choice is limited to a single volume. Needle-like crystals provide an additional dimension of complexity: to utilize the whole crystal volume a one-dimensional ‘scan’ across the length of the needle is desirable (Flot *et al.*, 2010 ▶) using a fixed beam size. However, plate-like crystals create a more complex challenge: to utilize the crystal volume efficiently two-dimensional scanning would be needed together with variation of the beam size dependent on crystal orientation. Such procedures may possibly have allowed data collection from a single SeMet crystal of rATX, which might in turn have enabled a more accurate recording of the anomalous signal to facilitate structure solution. In retrospect, the main obstacle to structure solution from these data was the size of the crystals rather than the diffraction limit or other basic problems.

Determination of the crystal structure of rATX was made possible by the addition of two components to the precipitating solution of our ‘basic’ crystallization protocol (Day *et al.*, 2010 ▶).

## Figures and Tables

**Table 1:** Sample information

	Crystal 1	Crystal 2	Crystal 3
Macromolecule details			
Mass (Da)	98000	98000	98000
Source organism	*Rattus norvegicus*	*Rattus norvegicus*	*Rattus norvegicus*
Crystallization and crystal data			
Crystallization method	Sitting-drop vapour diffusion	Sitting-drop vapour diffusion	Sitting-drop vapour diffusion
Temperature (K)	293	293	293
Crystal-growth time (d)	4–5	4–5	2
Crystallization solutions			
Macromolecule	4 µl 3–4 mg ml^−1^ rATX, 150 m*M* NaCl, 20 m*M* Tris–HCl pH 8	4 µl 3–4 mg ml^−1^ rATX, 150 m*M* NaCl, 20 m*M* Tris–HCl pH 8	200 nl 3–4 mg ml^−1^ rATX, 150 m*M* NaCl, 20 m*M* Tris–HCl pH 8
Precipitant	5 µl 20%(*w*/*v*) polyethylene glycol 6000, 100 m*M* potassium citrate, 100 m*M* MIB buffer pH 8.5	5 µl 18–22%(*w*/*v*) polyethylene glycol 6000, 100 m*M* potassium citrate, 100 m*M* MIB buffer pH 8.5	100 nl 20%(*w*/*v*) polyethylene glycol 3350, 100 m*M* bis-tris propane pH 6.5–8.5
Additive	1 µl 1 *M* MEGA-8/10	1 µl 1 *M* MEGA-8/10	20–200 m*M* sodium/potassium phosphate
Crystal data			
Crystal dimensions (µm)	∼100 × 50 × 1	∼150 × 30 × 1	∼200 × 30 × ∼1
Matthews coefficient *V*_M_ (Å^3^ Da^−1^)	2.38	2.38	2.38
Solvent content (%)	48	48	48
Unit-cell data			
Crystal system, space group	Orthorhombic, *P*2_1_2_1_2_1_	Orthorhombic, *P*2_1_2_1_2_1_	Orthorhombic, *P*2_1_2_1_2_1_
Unit-cell parameters (Å, °)	*a* = 95.3, *b* = 121.8, *c* = 158.4, α = β = γ = 90	*a* = 96.2, *b* = 121.7, *c* = 160.0, α = β = γ = 90	*a* = 95.4, *b* = 120.2, *c* = 157.1, α = β = γ = 90
No. of molecules in unit cell *Z*	2	2	2

**Table 2:** Data-collection statistics from individual crystals Values in parentheses are for the outer shell and those in square brackets are for that around 3.2 Å resolution (shown for better appreciation of the data-completeness issues).

	Crystal 1	Crystal 2	Crystal 3
Source	SLS X06SA	SLS X06SA	Diamond I24
X-ray beam size (µm)	10 × 10	40 × 100	8 × 8/15 × 20/30 × 50
Wedges collected (°)	0–30/40–60/70–90	0–25	0–10/10–50/60–100
Crystal-to-detector distance (mm)	300	520.0	535.9
Exposure time (s)	1	1	2
Rotation range per image (^o^)	1.0	0.25	1.0
Diffraction protocol	Single wavelength	Single wavelength	Single wavelength
Wavelength (Å)	0.933	0.978	0.978
Detector	MAR 225 CCD	Pilatus 6M	Pilatus 6M
Temperature (K)	100	100	100
Resolution range (Å)	50.0–2.6 (2.74–2.6) [3.36–3.11]	50.0–2.6 (2.74–2.6) [3.36–3.11]	50.0–2.6 (2.74–2.6) [3.36–3.11]
No. of unique reflections	46204 (3355)	30282 (3567)	48210 (3048)
No. of observed reflections	120917 (6162)	42546 (4112)	131664 (3748)
Completeness (%)	80.5 (40.2) [97.5]	52.9 (43.2) [58.8]	86.8 (38.6) [97.8]
Multiplicity	2.6 (1.8) [2.7]	1.4 (1.2) [1.4]	2.7 (1.2) [3.2]
〈*I*/σ(*I*)〉	4.9 (1.6) [3.1]	4.0 (1.4) [3.8]	4.5 (1.3) [3.7]
*R*_merge_	0.180 (0.511) [0.371]	0.106 (0.299) [0.180]	0.186 (0.364) [0.300]
Data-processing software	*MOSFLM*/*SCALA*	*XDS*/*SCALA*	*XDS*/*SCALA*

**Table 3:** Data-collection statistics for the combined data set Values in parentheses are for the outer shell and those in square brackets are for that around 3.2 Å resolution.

	Crystals 1 + 2
Source	SLS X06SA
Resolution range (Å)	50.0–2.6 (2.74–2.6) [3.36–3.11]
No. of unique reflections	52092 (5747)
No. of observed reflections	162655 (10544)
Completeness (%)	91.0 (69.7) [99.0]
Redundancy	3.1 (1.8) [3.4]
〈*I*/σ(*I*)〉	7.7 (1.5) [4.1]
*R*_merge_	0.191 (0.488) [0.359]
Data-processing software	*MOSFLM*/*SCALA*

## References

[bb1] Aishima, J., Owen, R. L., Axford, D., Shepherd, E., Winter, G., Levik, K., Gibbons, P., Ashton, A. & Evans, G. (2010). Submitted. 10.1107/S0907444910028192 PMC6691516 20823554

[bb2] Albers, H. M., Dong, A., van Meeteren, L. A., Egan, D. A., Sunkara, M., van Tilburg, E. W., Schuurman, K., van Tellingen, O., Morris, A. J., Smyth, S. S., Moolenaar, W. H. & Ovaa, H. (2010). *Proc. Natl Acad. Sci. USA*, **107**, 7257–7262. 10.1073/pnas.1001529107 PMC2867685 20360563

[bb3] Aricescu, A. R. *et al.* (2006). *Acta Cryst.* D**62**, 1114–1124.

[bb4] Beteva, A. *et al.* (2006). *Acta Cryst.* D**62**, 1162–1169. 10.1107/S0907444906032859 17001093

[bb5] Bowler, M. W., Guijarro, M., Petitdemange, S., Baker, I., Svensson, O., Burghammer, M., Mueller-Dieckmann, C., Gordon, E. J., Flot, D., McSweeney, S. M. & Leonard, G. A. (2010). *Acta Cryst.* D**66**, 855–864. 10.1107/S0907444910019591 20693684

[bb6] Broennimann, C., Eikenberry, E. F., Henrich, B., Horisberger, R., Huelsen, G., Pohl, E., Schmitt, B., Schulze-Briese, C., Suzuki, M., Tomizaki, T., Toyokawa, H. & Wagner, A. (2006). *J. Synchrotron Rad.* **13**, 120–130. 10.1107/S0909049505038665 16495612

[bb7] Cherezov, V., Hanson, M. A., Griffith, M. T., Hilgart, M. C., Sanishvili, R., Nagarajan, V., Stepanov, S., Fischetti, R. F., Kuhn, P. & Stevens, R. C. (2009). *J. R. Soc. Interface*, **6**, S587–S597. 10.1098/rsif.2009.0142.focus PMC2843980 19535414

[bb8] Cusack, S., Belrhali, H., Bram, A., Burghammer, M., Perrakis, A. & Riekel, C. (1998). *Nature Struct Biol.* **5**, 634–637. 10.1038/1325 9699611

[bb9] Day, J. E., Hall, T., Pegg, L. E., Benson, T. E., Hausmann, J. & Kamtekar, S. (2010). *Acta Cryst.* F**66**, 1127–1129. 10.1107/S1744309110030228 PMC2935245 20823544

[bb26] Evans, G., Alianelli, L., Burt, M., Wagner, A. & Sawhney, K. (2006). *AIP Conf. Proc.* **879**, 836-839.

[bb10] Flot, D., Mairs, T., Giraud, T., Guijarro, M., Lesourd, M., Rey, V., van Brussel, D., Morawe, C., Borel, C., Hignette, O., Chavanne, J., Nurizzo, D., McSweeney, S. & Mitchell, E. (2010). *J. Synchrotron Rad.* **17**, 107–118. 10.1107/S0909049509041168 PMC3025444 20029119

[bb11] Fotopoulou, S., Oikonomou, N., Grigorieva, E., Nikitopoulou, I., Paparountas, T., Thanassopoulou, A., Zhao, Z., Xu, Y., Kontoyiannis, D. L., Remboutsika, E. & Aidinis, V. (2010). *Dev. Biol.* **339**, 451–464. 10.1016/j.ydbio.2010.01.007 20079728

[bb12] Jansen, S., Callewaert, N., Dewerte, I., Andries, M., Ceulemans, H. & Bollen, M. (2007). *J. Biol. Chem.* **282**, 11084–11091. 10.1074/jbc.M611503200 17307740

[bb13] McCoy, A. J., Grosse-Kunstleve, R. W., Adams, P. D., Winn, M. D., Storoni, L. C. & Read, R. J. (2007). *J. Appl. Cryst.* **40**, 658–674. 10.1107/S0021889807021206 PMC2483472 19461840

[bb14] Meeteren, L. A. van & Moolenaar, W. H. (2007). *Prog. Lipid Res.* **46**, 145–160. 10.1016/j.plipres.2007.02.001 17459484

[bb15] Meeteren, L. A. van, Ruurs, P., Stortelers, C., Bouwman, P., van Rooijen, M. A., Pradere, J. P., Pettit, T. R., Wakelam, M. J., Saulnier-Blache, J. S., Mummery, C. L., Moolenaar, W. H. & Jonkers, J. (2006). *Mol. Cell. Biol.* **26**, 5015–5022. 10.1128/MCB.02419-05 PMC1489177 16782887

[bb27] Mills, G. B. & Moolenaar, W. H. (2003). *Nature Rev. Cancer*, **3**, 582–591. 10.1038/nrc1143 12894246

[bb16] Moukhametzianov, R., Burghammer, M., Edwards, P. C., Petitdemange, S., Popov, D., Fransen, M., McMullan, G., Schertler, G. F. X. & Riekel, C. (2008). *Acta Cryst.* D**64**, 158–166. 10.1107/S090744490705812X PMC2467531 18219115

[bb17] Newman, J., Egan, D., Walter, T. S., Meged, R., Berry, I., Ben Jelloul, M., Sussman, J. L., Stuart, D. I. & Perrakis, A. (2005). *Acta Cryst.* D**61**, 1426–1431. 10.1107/S0907444905024984 16204897

[bb18] Perrakis, A., Cipriani, F., Castagna, J.-C., Claustre, L., Burghammer, M., Riekel, C. & Cusack, S. (1999). *Acta Cryst.* D**55**, 1765–1770. 10.1107/s0907444999009348 10531527

[bb19] Sanishvili, R., Nagarajan, V., Yoder, D., Becker, M., Xu, S., Corcoran, S., Akey, D. L., Smith, J. L. & Fischetti, R. F. (2008). *Acta Cryst.* D**64**, 425–435. 10.1107/S0907444908001741 PMC2631116 18391409

[bb20] Schneider, T. R. (2008). *HFSP J.* **2**, 302–306. 10.2976/1.2982661 PMC2645582 19436492

[bb21] Song, J., Mathew, D., Jacob, S. A., Corbett, L., Moorhead, P. & Soltis, S. M. (2007). *J. Synchrotron Rad.* **14**, 191–195. 10.1107/S0909049507004803 17317920

[bb22] Stracke, M. L., Krutzsch, H. C., Unsworth, E. J., Arestad, A., Cioce, V., Schiffmann, E. & Liotta, L. A. (1992). *J. Biol. Chem.* **267**, 2524–2529. 1733949

[bb23] Tanaka, M., Okudaira, S., Kishi, Y., Ohkawa, R., Iseki, S., Ota, M., Noji, S., Yatomi, Y., Aoki, J. & Arai, H. (2006). *J. Biol. Chem.* **281**, 25822–25830. 10.1074/jbc.M605142200 16829511

[bb24] Umezu-Goto, M., Kishi, Y., Taira, A., Hama, K., Dohmae, N., Takio, K., Yamori, T., Mills, G. B., Inoue, K., Aoki, J. & Arai, H. (2002). *J. Cell. Biol.* **158**, 227–233. 10.1083/jcb.200204026 PMC2173129 12119361

[bb25] Zalatan, J. G., Fenn, T. D., Brunger, A. T. & Herschlag, D. (2006). *Biochemistry*, **45**, 9788–9803. 10.1021/bi060847t 16893180

